# Predicting Volleyball Serve-Reception

**DOI:** 10.3389/fpsyg.2016.01694

**Published:** 2016-11-02

**Authors:** Ana Paulo, Frank T. J. M. Zaal, Sofia Fonseca, Duarte Araújo

**Affiliations:** ^1^Faculdade de Motricidade Humana, CIPER, Universidade de LisboaLisbon, Portugal; ^2^Center for Human Movement Sciences, University Medical Center Groningen, University of GroningenGroningen, Netherlands; ^3^Faculdade de Educação Física e Desporto, Universidade Lusófona de Humanidades e TecnologiasLisbon, Portugal

**Keywords:** decision making, interceptive action, pass, expertise, sports, logistic regression

## Abstract

Serve and serve-reception performance have predicted success in volleyball. Given the impact of serve-reception on the game, we aimed at understanding what it is in the serve and receiver's actions that determines the selection of the type of pass used in serve-reception and its efficacy. Four high-level volleyball players received jump-float serves from four servers in two reception zones—zone 1 and 5. The ball and the receiver's head were tracked with two video cameras, allowing 3D world-coordinates reconstruction. Logistic-regression models were used to predict the type of pass used (overhand or underhand) and serve-reception efficacy (error, out, or effective) from variables related with the serve kinematics and related with the receiver's on-court positioning and movement. Receivers' initial position was different when in zone 1 and 5. This influenced the serve-related variables as well as the type of pass used. Strong predictors of using an underhand rather than overhand pass were higher ball contact of the server, reception in zone 1, receiver's initial position more to the back of the court and backward receiver movement. Receiver's larger longitudinal displacements and an initial position more to the back of the court had a strong relationship with the decreasing of the serve-reception efficacy. Receivers' positioning and movement were the factors with the largest impact on the type of pass used and the efficacy of the reception. Reception zone affected the variance in the ball's kinematics (with the exception of the ball's lateral displacement), as well as in the receivers' positioning (distances from the net and from the target). Also the reception zone was associated with the type of pass used by the receiver but not with reception efficacy. Given volleyball's rotation rule, the receiver needs to master receiving in the different reception zones; he/she needs to adapt to the diverse constraints of each zone to maintain performance efficacy. Thus, being able to flexibly vary positioning and passing, given local (zone) constraints, can yield an advantage in high-level volleyball serve-reception. Further, research needs to consider other serve modes (e.g., power-jump serve) and a full-court context of performance to support the present study's findings.

## Introduction

Serve and serve-reception (referred to as “reception” from this point on) performance have been identified as predictors of team success in volleyball (Peña et al., 2013; Silva et al., 2014). In volleyball's performance analysis literature, these two actions tend to be analyzed separately (e.g., Eom and Schutz, 1992; Coleman, 2002), which leaves unclear the impact of the server and receiver's contributions to the point being disputed (see Afonso et al., 2009). The present study considers the serve-reception from the perspective of the receiving player: What are the constraints that channel an effective reception? What influences the selection of the mode of action (type of pass) used for reception, and how does this selection influences passing efficacy? Characteristics of the serve play a role in the serve-reception efficacy. Less clear are the effects of the positioning and moving of the receiver. How do all these characteristics (constraints) combine?

A number of studies have indicated the characteristics of an effective serve. For instance, Deprá and Brenzikofer (2008) compared four age groups of volleyball players (from Under-13 to Under-20) with an expert player, studying the kinematic characteristics of the ball in four serve techniques. They found that higher velocities, lower ball heights when crossing the net, and smaller initial throw angles were the constraints of the serve that distinguished the expert player's serve from the other groups. Using both male and female adult level volleyball players, MacKenzie et al. (2012), targeting the jump-float serve technique, related better serving performance (harder constraints on serve-reception) with higher server's contact with the ball, higher initial velocity and a flatter projection angle. Both studies had the same limitation for the study of the serve efficacy: no actual reception occurred. On the other hand, Wang and Liu (2009) studied the relationship between the just mentioned kinematic characteristics and the success of the reception. They argued that the servers' movement as well as the trajectory and velocity of the ball need to be investigated as key information-sources for receivers to successfully perform the reception. So an effective serve challenges the receiver, but how do the ball cinematics constrain the reception outcome, remains unclear. Moras et al. (2008), who studied expert male players in an official competition, did not find any relationship between serve speed and reception efficacy. A relationship between serve-related factors and reception efficacy is to be expected because receivers rely on the kinematics of the server and of the serve itself, for information to realize their reception and subsequent passing (Lenoir et al., 2005; Wang and Liu, 2009). From an ecological dynamics approach (Araújo et al., 2006; Davids et al., 2015) not only the constraints of the ball approach but also those of the receiving player will determine how the serve will be handled by the receiver (action mode selection, i.e., type of pass used), and, as a consequence, the effectiveness of the pass to the setter.

Reception can be performed by an underhand or by an overhand mode of action (type of pass). The use of an overhand pass appears to increase the chances of an effective reception (Afonso et al., 2012). The decision to use one or the other pass seems to be related to serve-related factors as well as the receiver's position and displacement on the court (Miller, 2005; Barsingerhorn et al., 2013; Dunphy and Wilde, 2014). Barsingerhorn et al. (2013) in a passing task found that the overhand pass was more frequently used closer to the initial position of the passer and the underhand pass was more frequent when the passer had to perform larger longitudinal displacements, both to the front and to the back of the initial position. But where is the initial position of the receiver on the court? There are task-related constraints to playing volleyball that might influence the receivers positioning and movement. For example, in high-level the teams almost exclusively use the 5:1 system of play (one setter and five attackers) and three priority receivers (see for further detail USA Volleyball, 2009). In this type of organization the volleyball's rotation rule (see for further detail FIVB, 2014) leads to: (i) each receiver having to master reception in different zones of the court; and (ii) having to receive and be available to attack near the net when receiving in the left and right side of the court. Katsikadelli (1997) found that in a competitive setting more serves were directed to the right side (server's perspective) of the court. Two reasons of why this might be the case are that the right side is the side where the attacking-receiver is in five of the six possible rotations and that it is also the side where the receiver is further away from his target (i.e., the setting zone, see Afonso et al., 2012). Thus, there are probably functional differences associated with where on court the reception takes place that might also be relevant constraints on reception, and therefore to be taken into account when trying to understand the emergent behavior.

Given serve-reception's impact on the volleyball game we aimed at further develop its understanding. Following the ecological dynamics framework we studied the link between the serve's cinematics and its impact on the reception; this impact is constrained, we expected, by where on the court the reception takes place (reception zone), and the receiver's on-court positioning and movement, with consequences to the selection of the receiver's type of pass, and on reception efficacy.

## Materials and methods

### Sample

Eight right-handed male expert volleyball players aged 27 ± 2.8 years (Mean ± *SD*) and with 15 ± 4.2 years of practice, all with international-level experience, participated in the study. Four of the players were servers and four were expert receivers in the runner-up team of the Portuguese first league. The study was approved by the Ethics Council of the Faculty of Human Kinetics, University of Lisbon (Nb. 7/2014). It was found to be in accordance with Portuguese and international guidelines for scientific research involving humans, including the 2013 Declaration of Helsinki on Ethical Principles for Medical Research Involving Human Subjects, and the 1997 Convention on Human Rights and Biomedicine (the “Oviedo Convention”).

### Task

**Figure 1:**
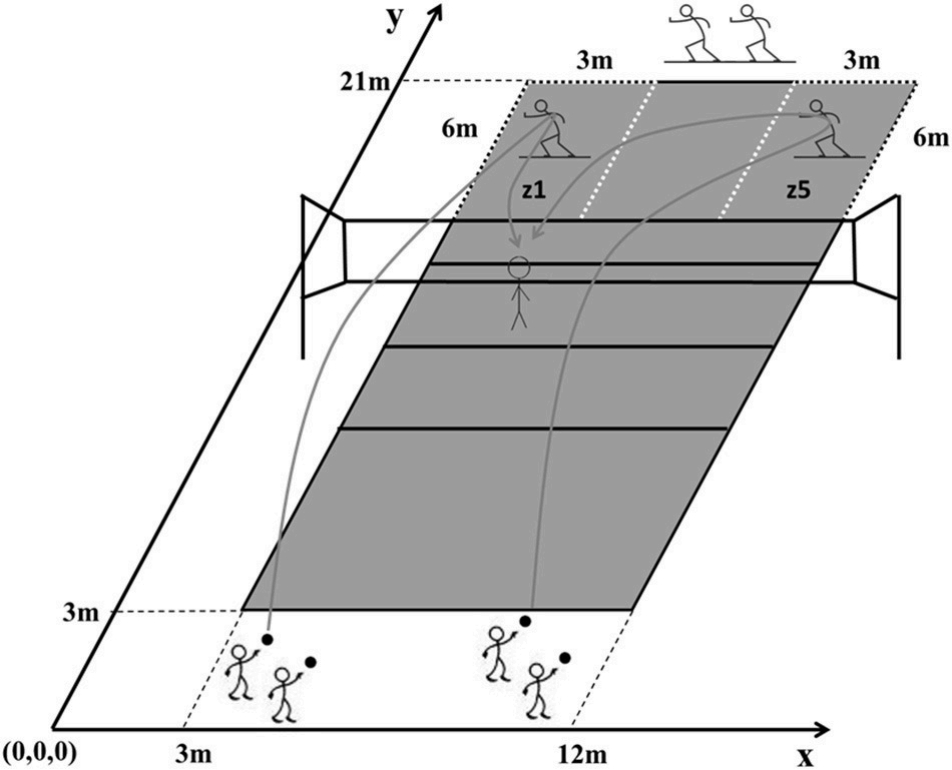
**Experimental set-up**. The two reception zones are labeled as z1 (zone 1) and z5 (zone 5).

The servers were positioned behind the back-line on one half of a volleyball court, two on the right side and two on the left side of the court (see Figure 1). Two reception areas were used: zones 1 and 5 (see FIVB, 2014). The receivers waited behind the back line for their turn. For each trial, one receiver entered one of the reception areas, performed the reception, and returned behind the backline to be ready to enter the other reception area. The servers on the right side delivered jump-float serves to zone 1 and the servers on the left side to zone 5 (receivers' perspective). Halfway the session, the servers changed side (left-to-right and vice-versa). A setter was positioned in the setting area (near the net) to play, whenever possible, the balls passed by the receivers. The servers were instructed to try to make a point by serving or to hinder the receiver's actions. The receivers were instructed to try to pass the ball to the setter in the best possible way. The session lasted 15 min in total, resulting in a sample of 136 reception trials (see Supplemental Task Video).

### 3D reconstruction

Two cameras were used to record the serves and their receptions at a frame rate of 25 Hz. Positions of the ball and receiver's head were obtained from the recorded videos. First, Labbio62.15 software (an updated version of TACTO, see Serrano and Fernandes, 2011) was used to determine 2D camera coordinates. Next, 3D world coordinates were computed from these 2D camera coordinates, using DLT algorithms programmed in MATLAB R2009a (Reinschmidt, January 1994; September 1994). The measurement volume was calibrated using 49 reference points (spanning a 12 × 21 × 3 m volume) and video recordings were synchronized on the basis of the moment that the server contacted the ball.

To estimate the error associated with the process of attaining 3D coordinates used, the 2D coordinates of the digitized calibration points were inserted into the 3D reconstruction MATLAB program, and the resulting coordinates (x, y, and z) were compared to the real (known) ones. The median error was 8 cm with an interquartile range (IQR) of 7 cm and a maximum error of 37 cm (one data point). To assess intra-observer reliability, the same researcher digitized twice the ball trajectory of 6 trials. After running the 2D coordinates through the 3D-reconstruction MATLAB script, differences between the two trials were determined. This resulted in a median difference of 7 cm (IQR of 5 cm). Since footage from two cameras was used for the 3D reconstruction, it was expected an additional error from discrepancies in the in synchronization of the cameras, which was not estimated. Smoothing splines (smoothing factor of 0.995) were applied to the reconstructed 3D positions of the ball before computing velocities and the other variables that entered the prediction model detailed next.

### Variables

A number of predictor-variables were considered for the logistic-regression models. Serve receptions occurred either in zone 1 or in zone 5. Serve-related variables were: flight time, initial ball velocity, projection angle, maximum height, longitudinal and lateral displacement, and height at server contact. In logistic regression models, *Exp*(ß_*i*_) represents the odds-ratio of success vs. failure (categories of the model's dependent variable) when variable *X*_*i*_ increases by one unit with respect to the odds-ratio of success vs. failure when *X*_*i*_ stays constant. Therefore, the unit chosen to express the variable and its intrinsic variability affect its expression in the model. For this reason, serve's height at server contact and maximum height were presented in decimeters, since in both variables the values' range was below 1 m. Receiver-related variables were initial position (i.e., distance from the net at server's ball contact), longitudinal and lateral displacements, and a categorical variable, front-back displacement that coded for the direction of movement: longitudinal displacement could be backward (moving away from the net) or forward (approaching the net). Other receiver-related variables were the longitudinal, lateral, and linear distance to the target at the moment of reception. The center of the “excellent setting zone” was taken as target (see Afonso et al., 2012).

As for the to-be-predicted variables, the types of pass considered were the *overhand pass* (fingertips contact ball above the head) and the *underhand pass* (forearm contact ball below the head). Three categories of reception efficacy, adapted from existing coding schemes (Afonso et al., 2012; Ciuffarella et al., 2013), were used: *error*, when receiver's contact doesn't allow setting, or restricts the setting options to one; *out*, when the setter has to set outside the excellent setting zone, or in the setting zone, but without all setting options; and, *effective*, when the reception allows setting in the excellent setting zone with all setting options available to the setter. For reception efficacy, type of pass was also a considered as predictor variable.

### Analysis

The predictor variables were initially described (mean and standard deviation) and analyzed with respect to the effect of the manipulated condition on the study—reception zone. Effect sizes were calculated following recommendations by Ferguson (2009) and Lakens (2013).

Logistic regression was used to develop two models that related ball and receiver kinematics with the type of pass (binomial model) and the reception efficacy (multinomial model), respectively. In developing the logistic-regression models, initially, all variables were included. Next, a manual backward stepwise procedure was performed, in which variables that did not contribute to the best predictive model were removed. In selecting a final model the following points were considered (Tabachnick and Fidell, 2013): (i) quality of the adjusted model; (ii) improvement of case classification by the model over classification by chance; (iii) goodness-of-fit criteria (Hosmer and Lemeshow Test); (iv) correlation of estimates to screen for multicollinearity problems (bivariate correlations > 0.90 considered indicative of collinearity problems); (v) the standard error of ß (used to screen for problems in a variable's expression in the model); (vi) odds-ratio values of the predictors; (vii) case wise listing of residuals for identification of outliers, defined as residuals larger than two standard deviations. Significance level was set at 0.05. The odds-ratio effect size was evaluated using values of 1.52 (small), 2.74 (medium), and 4.72 (large) as evaluation criteria (Chen et al., 2010).

## Results

Serves were delivered either to the left side (zone 5) or the right side (zone 1) of the court (see Figure 1). *T*-tests indicated slight differences between sides in the characteristics of the serves and of the receivers' motion (see Table 1). Compared to zone 5, serves arriving in zone 1, on average, had a significantly shorter flight time [1.02 (zone 1) vs. 1.08 s (zone 5), *t* = −2.730, *p* = 0.007], higher initial velocity (16.36 vs. 14.54 m/s, *t* = 6.641, *p* <0.001), lower maximum height (2.60 vs. 2.81 m, *t* = −9.147, *p* <0.001), longer longitudinal displacement (15.91 vs. 14.64 m, *t* = 8.479, *p* <0.001), lower height at server contact (2.50 vs. 2.64 m, *t* = −10.711, *p* <0.001), and a smaller projection angle (1.63 vs. 2.69°, *t* = −3.986, *p* <0.001). Despite their statistical significance, the reception zone had only a medium effect size on the serve's flight time and projection angle's. The reception zone effect was large for the other serve-related variables. Regarding the receivers, in zone 1 their initial position was farther from the net (6.96 vs. 6.69 m, *t* = 5.020, *p* <0.001), their longitudinal distance to the target was larger (5.74 vs. 5.26 m, *t* = 4.006, *p* <0.001), and their lateral and linear distance to the target were smaller (1.50 vs. 3.51 m, *t* = −14.533, *p* <0.001, and 5.99 vs. 6.35 m, *t* = −2.713, *p* = 0.006, respectively). The reception zone had a large effect in the receiver-related variables, with the exception of the longitudinal (medium effect) and linear (small effect) distances to the target.

**Table 1:** **Characterization of serve and receiver's potential predictor-variables**.

			**Overall**	**Reception zone**		**Test value (*t* or χ^2^)**	***p***	**Effect size (Hedges's g_s_ or Cramer's V)**
			***n* = 136**	**zone 1** ***n* = 74**	**zone 5** ***n* = 62**			
Serve (Ball)	Flight time (s)		1.05±0.11	1.02±0.11	1.08±0.11	−2.730^δ^	0.007^δ^	0.54^δ^
	Initial velocity (m.s^−1^)		15.53±1.83	16.36±1.70	14.54±1.46	6.641^δ^	<0.001^δ^	1.13^δ^
	Maximum height (dm)		26.95±1.65	26.02±1.26	28.06±1.35	−9.147^δ^	<0.001^δ^	1.56^δ^
	Displacement	Longitudinal (m)	15.33±1.08	15.91±0.92	14.64±0.81	8.479^δ^	<0.001^δ^	1.45^δ^
		Lateral (m)	0.93±0.63	0.88±0.59	0.98±0.66	−0.975^δ^	0.332^δ^	0.16^δ^
	Height at server contact (dm)		25.63±0.97	25.02±0.70	26.35±0.74	−10.711^δ^	<0.001^δ^	1.84^δ^
	Projection angle (°)		2.11±1.62	1.63±1.51	2.69±1.58	−3.986^δ^	<0.001^δ^	0.68^δ^
Receiver	Initial position (m)		6.84±0.34	6.96±0.32	6.69±0.30	5.020^δ^	<0.001^δ^	0.86^δ^
	Displacement	Longitudinal (m)	0.44±0.43	0.40±0.36	0.48±0.51	−1.070^δ^	0.287^δ^	0.18^δ^
		Lateral (m)	0.57±0.43	0.58±0.44	0.56±0.42	0.244^δ^	0.807^δ^	0.05^δ^
	Front-back displacement	Back [*n*(%)]	52 (38.5%)	27 (36.5%)	25 (41%)	0.286	0.593	0.05^†^
		Front [*n*(%)]	83 (61.5%)	47 (63.5%)	36 (59%)			
	Distance to target	Longitudinal (m)	5.52±0.74	5.74±0.65	5.26±0.76	4.006^δ^	<0.001^δ^	0.68^δ^
		Lateral (m)	2.42±1.28	1.50±0.83	3.51±0.76	−14.533^δ^	<0.001^δ^	2.50^δ^
		Linear (m)	6.16±0.77	5.99±0.65	6.35±0.86	−2.713^δ^	0.008^δ^	0.48^δ^

*Continuous data are reported as mean ± SD*.

*Categorical data are reported as n (%)*.

δ Independent samples T-test, Hedges's g_s_;

† *Chi-square test, Cramer's V*.

*In one trial the receiver's initial and final position did not change, and therefore the front-back displacement variable has one missing case*.

Pearson χ^2^ tests showed that the distributions of the type of pass used in reception were different in the two reception zones [$\chi_{\left(1,\;\text{N}\;=\;136\right)}^{2}$ = 5.902, *p* = 0.015, Φ_Cramer_ = 0.21]. The overhand pass (*N* = 29) was used more frequently in zone 5 (19 out of the 62 passes in this zone; 30.6%) than in zone 1 (10 out of 74 passes; 13.5%), whereas the underhand pass (*N* = 107) was more prevalent in zone 1 (*N* = 64, 86.5%), than in zone 5 (*N* = 43, 69.4%). No associations were found between reception zone and reception efficacy [$\chi_{\left(2,\;\text{N}\;=\;136\right)}^{2}=$ 0.672, *p* = 0.715] nor between the type of pass used in reception and reception efficacy [$\chi_{\left(2,\;\text{N}\;=\;136\right)}^{2}$ = 0.462, *p* = 0.794].

To arrive at the predictive model of type of pass, first, all potential predictor-variables (see Table 1) were entered, and next, step by step, variables that caused problems of multicollinearity (flight time, projection angle, and linear distance to the target) or that did not add significantly to the predictive power of the model (serve: initial velocity; receiver: longitudinal and lateral displacement, and longitudinal and lateral distance to the target) were removed. Because front-back displacement was not present in one trial, we removed this trial from the data set, leaving 135 trials for analysis. Four outliers were identified and also removed. The remaining 131 trials included 26 overhand passes (19.85%) and 105 underhand passes (80.15%).

The final model performed significantly better than a constant-only model [$G_{\left(7,\;N\;=\;131\right)}^{2}$ = 65.010, *p* <0.001] and satisfied goodness-of-fit criteria [Hosmer and Lewenshow test: $\chi_{\left(8,\;\text{N}\;=\;131\right)}^{2}$ = 9.206, *p* = 0.325], resulting in a Nagelkerke *r*^2^ of 0.62. There was a correct classification of 89.3% of the cases, 17 out of the 26 overhand passes (65.4%) and 100 out of the 105 underhand passes (95.2%).

As can be seen in Table 2 the odds for using an underhand rather than overhand pass increased when the server contacted the ball at higher points, the serve reached a lower maximum height, and had smaller longitudinal and lateral displacements. Also, the odds for the underhand pass use increased when the receiver's initial position was more to the back of the court, and when he moved backward. In addition, the odds for underhand pass use were higher in zone 1 than in zone 5. All receiver-related variables, as well as the reception zone and server's height of contact had a large effect on the model.

**Table 2:** **Final binary logistic regression model of type of pass**.

			**ß (*S.E*.)**	**χ^2^**	***p***	**Exp(ß)**	**Exp(*ß*) 95% CI**	
							**Lower**	**Upper**
Receiver	Initial position (m)		5.871 (1.278)	21.096	<0.001	354.540	28.951	4341.797
	Forward−Backward displacement	(Backward)	3.307 (1.113)	8.834	0.003	27.315	3.084	241.897
Reception zone (zone 1)			1.817 (1.147)	2.510	0.113	6.152	0.650	58.207
Serve	Height at server contact (dm)		1.571 (0.539)	8.478	0.004	4.809	1.671	13.843
	Maximum height (dm)		−0.948 (0.333)	8.084	0.004	0.388	0.202	0.745
	Displacement	Longitudinal (m)	−0.883 (0.484)	3.332	0.068	0.414	0.160	1.067
		Lateral (m)	−0.508 (0.549)	0.859	0.354	0.601	0.205	1.762
Constant			−110.790 (26.957)	16.891	<0.001			

*Underhand pass is the reference category of type of pass predicted in the model*.

*For categorical variables, the reference category included in the model is identified in brackets*.

The final model for reception efficacy is given in Table 3. The total sample of 136 receptions was available for analysis: 49 *error* receptions (36.0%), 33 *out* receptions (24.3%), and 54 *effective* receptions (39.7%). The model performed significantly better than a constant-only model [$G_{\left(7,\;N\;=\;132\right)}^{2}$ = 35.501, *p* <0.001] and satisfied goodness-of-fit criteria [Hosmer and Lewenshow test: $\chi_{\left(260,\text{N}\;=\;136\right)}^{2}=$ 280.19, *p* = 0.186], resulting in a Nagelkerke *r*^2^ of 0.26. The model correctly classified 32 of the 49 *error* receptions (65.3%), 8 of the 33 *out* receptions (24.2%), and 42 of the 54 *effective* receptions (77.8%). There was an overall correct classification of 60.3% of the cases.

**Table 3:** **Final multinomial logistic regression model of reception efficacy**.

			**ß (*S.E*.)**	**χ^2^**	***p***	**Exp(*ß*)**	**Exp(*ß*) 95% CI**	
							**Lower**	**Upper**
Error	Receiver	Initial position (m)	1.324 (0.715)	3.433	0.064	3.759	0.926	15.252
		Longitudinal displacement (m)	2.361 (0.735)	10.312	0.001	10.600	2.509	44.782
		Lateral distance to target (m)	0.859 (0.252)	11.618	0.001	2.360	1.440	3.868
	Serve	Initial velocity (m.s^−1^)	0.810 (0.208)	15.217	<0.001	2.248	1.496	3.377
		Lateral displacement (m)	0.118 (0.413)	0.081	0.776	1.125	0.500	2.529
	Constant		–40.863 (14.908)	7.513	0.006			
Out	Receiver	Initial position (m)	2.543 (0.848)	8.988	0.003	12.713	2.412	67.016
		Longitudinal displacement (m)	0.036 (0.776)	0.002	0.963	1.037	0.227	4.743
		Lateral distance to target (m)	0.660 (0.258)	6.545	0.011	1.935	1.167	3.210
	Serve	Initial velocity (m.s^−1^)	0.261 (0.207)	1.590	0.207	1.299	0.865	1.949
		Lateral displacement (m)	0.695 (0.431)	2.602	0.107	2.004	0.861	4.661
	Constant		–54.692 (16.970)	10.386	0.001			

*Effective reception is the reference category of reception efficacy predicted in the model*.

In the final multinomial model, the odds of an *error* and an *out* reception occurring instead of an *effective* reception, increased with: a higher initial velocity and larger lateral displacement of the serve; receiver's initial position more to the back of the court, larger longitudinal displacement, and larger lateral distance to the target. In terms of effect size, the receiver's longitudinal displacement had a large effect on predicting an *error* rather than an *effective* reception; the receiver's initial position had a large effect on predicting an *out* rather than an *effective* reception.

## Discussion

The quality of reception is an important factor in winning a volleyball match (Peña et al., 2013; Silva et al., 2014). For practicing reception, an understanding of what determines reception efficacy is relevant. Using data collected from high-level players in a real practice setting, a large number of factors related with the serve itself and related with the receiver were scrutinized for their effects on the type of pass used and reception efficacy.

**Figure 2:**
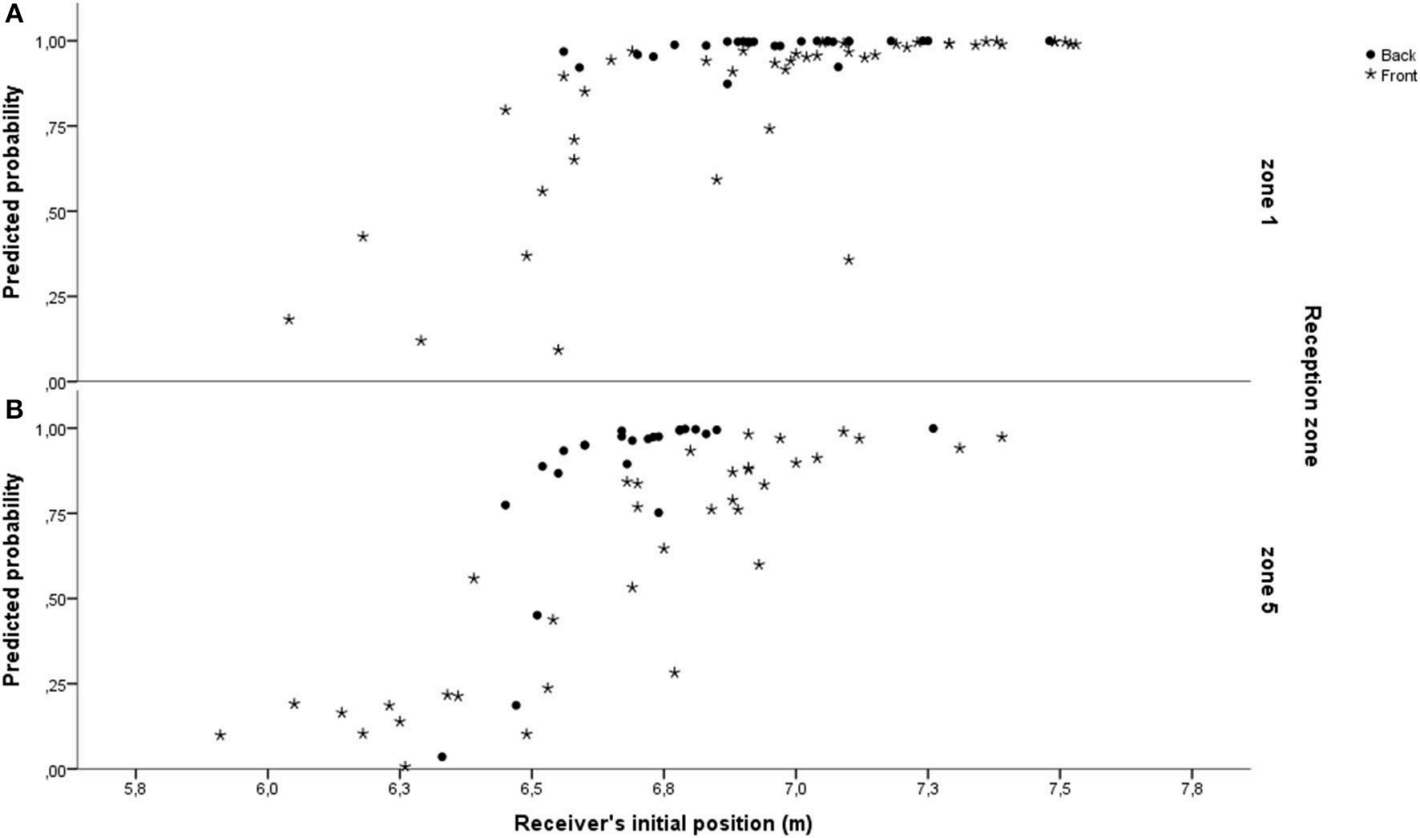
**Depiction, case-by-case, of the relation of the modeled type of pass prediction with the receiver's initial position (distance from the net)**. In the Y axis “1” corresponds to predicting the underhand pass and “0” to predicting the overhand pass. Cases are labeled by the direction of the receiver's displacement (to the front or to the back) and paneled by reception zone—zone 1 **(A)** and zone 5 **(B)**.

Based on previous studies (e.g., Katsikadelli, 1997; Barsingerhorn et al., 2013), there are task-related functional constraints in the reception that might influence the receiver's behavior. In our experimental design we expressed these constraints by considering two reception zones. The results of this study supported this claim since there was an influence of zone in every variable considered with respect to the serve and to the receiver, with the exception of the serve's lateral displacement and the receiver's displacement-related variables. For the serve's lateral displacement, this lack of influence might be related with the smaller lateral range of the reception zones (3 m) as opposed to their longitudinal range (6 m). As for the receiver's displacements, they are highly limited by the task's temporal constraints; the jump-float serve takes about 1 s to reach the receiver, who needs about 300 ms to initiate his first movement (see Benerink et al., 2015). In terms of the outcome variables, the type of pass used was associated with the reception zone but the reception efficacy was not. The overhand pass was used more frequently in zone 5, whereas the underhand pass was more prevalent in zone 1. We interpret these results in light of the ecological dynamics framework where the type of pass used is a way to deal with this local constraints (reception zone), allowing the receiver to keep efficacy levels in the two zones (Hristovski et al., 2006). This interpretation is reinforced by the fact that the reception zone was one of the predictors of the type of pass used. Receiving in zone 1 increased the odds of the receiver using the underhand pass. In the prediction of the type of pass used in reception, the most important factor, related with the serve itself, turned out to be the height at which the server hit the ball; the higher this point the higher the chances of an underhand reception. However, the odds of a serve leading to an underhand reception were more strongly influenced by the receiver's initial position on the court and his direction of movement. The farther the initial position from the net the higher the chances of taking the serve with an underhand pass. The chances of an underhand reception were also higher when the receiver had to move backward to intercept the approaching ball. Figure 2 shows a case-by-case relationship between the receiver's initial position and the model's predicted probabilities of type of pass used, constrained by the directionality of the receiver's longitudinal displacement, and by the reception zone. In Figure 2 we see the influence of the receiver's initial position with the predicted probabilities of the type of pass used; positions more to the back of the court, from 6.5 m, lead to a strong probability that the underhand pass was used. As for the directionality of the displacement, moving to the back relates with the use of the underhand pass, again from distances to the net above 6.5 m. We can also see that more cases with positions closer to the net were found in zone 5, and related with the model predicting them to the use of the overhand pass. Zone had an influence on the positioning of the receivers. On average, players in zone 5 were positioned slightly (but significantly) closer to the net than in zone 1. Why might this have been the case? Perhaps real game conditions carried over to the experimental task played a role. Whereas a practice situation such as the one that was used does not include any incentive toward this difference in positioning, a real game of volleyball does. In zone 5, the receiver is usually an attacking-receiver (in five out of the six possible rotations). The high speed of the attack of expert males (García-de-Alcaraz et al., 2015) makes the subsequent attack a possible additional constraint on the receiver, leading him to take an initial position closer to the net in zone 5, and positioning himself more to the front. Given that an initial position located more to the front increases the chances of the use of an overhand pass, this functional aspect of receiving in zone 5 might prompt the emergence of the overhand pass, being a possible explanation for its increased frequency in that zone. Since action-mode selection was free (no manipulation was imposed from the design), the emergent action mode was most probably due to serve and receiver-related constraints (Araújo et al., 2006; Davids et al., 2015) pending on local constraints, the reception zone (Hristovski et al., 2006). There was a large asymmetry in this sample with respect to the type of pass used. The overhand pass was only used in one-fifth of the times. This asymmetry is congruent with high-level male competitive settings, where the use of the overhand pass in reception has been reported as 6.5% of the full sample (Palao et al., 2009). This suggests that the solicitation to use the overhand pass might be more present when receiving in zone 5 than in zone 1. It is not an emergency technique, as has been suggested (Dunphy and Wilde, 2014), but it might be a way to better adjust to the requirements of this fast-paced sport, given the jump-float serve constraints (Shondell, 2002), and potentially having to attack after reception.

The differences in the initial positioning of the players in the two zones led to other consequences. First, serves were different for the two zones. Serves to zone 5, on average, were slower and with higher maximum height than those directed at zone 1. This suggests that a defending team might nudge servers toward preferred serves, a phenomenon that has been established in penalty kicks in football (Masters et al., 2007), and also in beach-volleyball (Noël et al., 2016). The server, tactically, “attacks” a player (e.g., least effective receiver in the opposing team) or he attacks court's “free space.” Given that the task under study was at the individual level, the receiver's different initial positioning freed up space differently in the two zones, arguably leading to different serves. All in all, the results suggest that how the receiver's positions himself on the court affects, to some extent, the details of the serve, but certainly how (type of pass used) he will probably handle the serve.

The type of pass used was not associated with reception efficacy. Using one pass or the other did not influence the odds of success, in contrast to the findings of Afonso et al. (2012). Since the type of pass used was predicted by the reception zone, but the later did not differ in terms of reception efficacy, the pass selection seems to have been a way to deal with each reception zone functional constraints so as to keep efficacy levels (Hristovski et al., 2006).

We were able to predict reception efficacy, having as predictors both details of the serve and of the receiver, to some extent supporting the ecological dynamics framework, where the behavior must be explained at the individual-environment level (Araújo et al., 2006; Davids et al., 2015). The receiver's initial position and also the amount of movement needed to intercept the ball were the most important factors on predicting reception efficacy. The distance to the target was a relevant factor on reception efficacy, particularly its lateral component; larger distance to the target increased significantly the odds of occurring an error and an out reception. There is interesting information in the model, from a performance analysis point of view, for the person delivering the serve: higher initial velocity, as suggested already in the literature (Deprá and Brenzikofer, 2008; Wang and Liu, 2009; MacKenzie et al., 2012) and forcing the receiver to considerable longitudinal movement will increase the odds of an error. Knowing the effects of these factors might prove helpful to the receiver—e.g., choosing an initial position closer to the net. However, the receiver has to deal with more constraints than simply being in the best position to receive the serve. His (relative) position is also determined by the rotation rule and considerations with respect to following attack options. For instance the latter might lead to receivers being position closer to the net, but at the same time further away from the target in zone 5. Still, being able to adapt positioning and displacement, within the latitude allowed by these constraints, may contribute to reception efficacy, and, as a consequence, to the chances of a positive game outcome.

The present study's findings indicated that the reception performance, in terms of action mode (type of pass) selection and its efficacy, is predicted by several factors related to the context of the performance—the serve, the receiver, and the reception zone. A fragmented approach where the serve and reception performances are not studied as an intertwined phenomenon limits the understanding of the game (Araújo and Davids, 2016). Our results suggest that the action mode that is selected might be a way to deal with local constraints in order to keep performance levels, as already suggested by Barsingerhorn et al. (2013) in a passing task. If so, practice should focus on potentiating the receiver's ability to adapt to the context of performance (through manipulation of the identified relevant factors) in order to develop flexibility in action mode (type of pass) selection. This approach to practice diverges from what is recommended in coaching literature (e.g., Hebert, 2014), which has a tendency to emphasize the stabilization of an ideal movement pattern in one-preferred solution type of tasks, usually technically centered.

As a final note, future studies may consider additional constraints to those considered in this study, which are relevant to reception performance as it occurs in a match. For instance, considering other serve types (most notably the power-jump serve), extending the reception area to the full-court, as well as the potential constraint of receiving with others, seem important next steps in this program of research.

## Conclusion

The quality of reception is an important predictor of team success. Several factors affect reception. The study of high-level volleyball players in a practice setting led to a better understanding of how the many factors that are involved interact in their effects on an effective reception. The positioning and movement of the defending players seem to have an effect on the details of the serve. The combined effects of serve details and of the positioning and movement of the receiver play out in the odds of the pass that the receiver used and of the efficacy of this pass. Interestingly, the type of pass used did not have an effect on reception efficacy. That is to say, whereas the receiver's position and movement have effects on how to pass a serve, these high-level players are able to flexibly adapt to varying constraints to keep up their level of performance. Practicing this flexibility might be the road to high-level performance in volleyball.

## Author contributions

AP, FZ, and DA made substantial, direct and intellectual contribution to the work, and approved it for publication. AP, FZ, and SF made substantial contributions to the analysis and the interpretation of the data. SF also gave final approval of the version to be published.

## Funding

This work was supported by the Portuguese Foundation for Science and Technology under Grant SFRH/BD/68692/2010 awarded to the first author.

### Conflict of interest statement

The authors declare that the research was conducted in the absence of any commercial or financial relationships that could be construed as a potential conflict of interest.
